# Design and Validation of a Grasping Force Measuring Vibrotactile Feedback Add-On for Laparoscopic Instruments

**DOI:** 10.1109/JTEHM.2025.3638856

**Published:** 2025-11-28

**Authors:** Jan-Willem Klok, Yannick Smits, Roelf Postema, Asþor T. Steinþorsson, Jenny Dankelman, Tim Horeman

**Affiliations:** Department of BioMechanical EngineeringDelft University of Technology 2860 Delft 2628 CD The Netherlands; Summox Dental B.V. Eindhoven 5642 JC The Netherlands; Spijkenisse Medisch Centrum Spijkenisse GA 3200 The Netherlands; Reon Reykjavik 105 Iceland; Amsterdam Skills Centre Amsterdam 1105 BD The Netherlands

**Keywords:** Compliant mechanisms, grasping, haptic feedback, laparoscopic surgery, postoperative complication prevention.

## Abstract

Objective: Grasping force control is crucial for safe laparoscopic surgery. However, force feedback is limited as haptic information on grasping strength and tissue stiffness is mostly lost due to internal instrument backlash and friction. This increases tissue trauma risk as excessive grasping forces can lead to (postoperative) complications. This study aims to develop a grasping force feedback providing add-on for a laparoscopic grasper and to validate its impact on skills acquisition in basic laparoscopic skills training. Method: The ShaftFlex, a shaft-based grasping force measurement system providing feedback was designed as an add-on for standard reusable instruments. It consists of a compliant element deflecting proportionally to the applied grasping force, and a Hall sensor measuring that deflection. Influence on skills acquisition was evaluated in a comparative study where novices were divided into a Feedback and No feedback group, performing five training trials of a silicon torus transfer boxtrainer task. Afterwards, both groups performed a post-training task without feedback. Grasping force, time to completion and number of errors were measured. Results: There was a significant difference in mean grasping force between groups for all training trials and the post-training trial. In the Feedback group, there was no significant increase in grasping force when feedback was removed. Conclusion: The ShaftFlex working principle provided a feasible, sustainable method to measure grasping forces exerted by a laparoscopic grasper, enabling immediate haptic feedback. It potentially enhances objective skill assessment, providing feedback on training performance. In a clinical context, the ShaftFlex might be useful in surgery where delicate tissue is grasped.

Clinical and Impact: This paper presents a compliant monolithic mechanism that enables direct vibro-tactile grasping force feedback in laparoscopic grasping instruments. It has the potential to reduce excessive grasping forces, therefore reducing intra- and post-operative complications. Moreover, it can be used as a tool to enhance learning in surgical novices.

## I. Introduction

Laparoscopy is a surgical procedure in which surgeons operate through small openings in the body using slender, specialized tools. These tools serve a variety of functions such as providing vision, cutting, grasping and stapling. The benefits of laparoscopy are evident as there is less scarring, shorter operation times and less postoperative pain. Also, there is lower risk of an inflammatory response and better visualization of inaccessible areas [1]. However, compared to open surgery, there are some drawbacks, as haptic information on grasping strength and tissue stiffness is mostly lost due to backlash and friction in the instrument [2], [3]. Other factors disturbing haptic feedback are trocar friction, mechanical efficiency of laparoscopic instruments, abdominal wall resistance, impeding hand-eye coordination and range of motion, and scaling due to the fulcrum effect [4], [5], [6]. Therefore, the learning curve for skills that are required to perform safe laparoscopic surgery is longer compared to open surgery [7].

In Laparoscopic surgery, grasping and other tissue interaction forces are often misjudged due to the lack of haptic information. Weber’s law states that the just noticeable difference of a stimulus is proportional to the magnitude of the original stimulus [8]. The (grasping) forces between tool tip and tissue are relatively small. In order to ensure a firm grip, surgeons sometimes apply much larger grasping forces than technically needed [9], exposing the patient to the risk of tissue damage [6], [10]. A study of Heijnsdijk et al. indicated that in some cases the grasping force is at least two times higher than necessary [11]. During laparoscopic cholecystectomy, approximately 25% of gallbladder perforations are caused by grasper trauma [12] while the occurrence of perforation can be 13% to 40% [13]. For efficient tissue manipulation under traction, repeated clamping and traumatic forces seem necessary to prevent slipping of the tissue. During a series of cholecystectomies, it was observed that grasped tissue is kept on traction for a relatively long time and incidences of tissue slip are frequent. Therefore it was argued that the design of laparoscopic graspers is not optimal for this type of frequently performed tasks [14]. Surgeons are less concerned with the damaging of tissue that will be removed when the risk of postoperative complications stemming from perforation is considered low. However, it has been shown that postoperative hospitalization is increased and infections at the ileus and trocar region infection occurred more often in patients with a gallbladder perforation or iatrogenic bowel trauma [13]. Therefore, for certain types of laparoscopic surgery a case can be made to improve or enhance grasping force feedback of instruments.

In scientific literature, numerous endeavors have already been made to reinstate haptic force feedback by gauging the forces within the instruments and subsequently conveying this information through a range of force feedback modalities. Most designs focus on integrating sensor technology in the instrument tip [15], [16]. The Force Reflecting Operation Instrument (FROI) measures forces using optical fiber Bragg technology and feeds back force information through a resistance mechanism in the handle [4]. Evaluation in a porcine study suggested that the instrument reduces grasping forces [17]. In another study, grasping force was measured in the tip and feedback was visually represented [18]. However, the in vitro study showed that sensor repeatability was low. A feedback-enabled laparoscopic demonstrated a promising compact design, and evaluation in a porcine model suggested a reduction in grasping force [19], although this effect was not statistically significant. A recent study proposed a promising sensing system by integrating force and angle sensors in the handle of laparoscopic graspers, providing visual feedback on tissue properties. Validation showed good correlation with jaw forces and improved task performance in preclinical tests [20]. In robotic surgery, feedback systems were developed by e.g. Asensus and Intuitive Surgical and integrated into their latest surgical systems. In one study, a force sensor and an augmented (visual) force representation system were used to provide grasping force feedback, which reduced grasping forces during a pick and place task [21].

These examples show that surgeons and engineers are aware of the relevance of reintroducing haptic grasping force feedback in minimally invasive surgery. However, in literature most proposed designs intended for laparoscopic surgery are technically complex, especially when attempts are made to integrate sensors in the small instrument tip [16], [17], [19]. This hampers introduction into surgery. In addition, little is known about the clinical effects and risks of switching between systems that provide haptic feedback and systems without. Therefore, the aim of this study is to develop a relatively simple grasping force feedback add-on for a laparoscopic grasper and to validate its impact on skills acquisition in basic laparoscopic skills training. The hypothesis is that during training, grasping with force feedback will reduce the grasping force compared to grasping without force feedback. Also, it is investigated when grasping force feedback is removed after training with force feedback, whether users will exert higher grasping forces compared to users that trained without force feedback.

## II. Methods

The Bare Minimum Design method with a focus on modularity and complexity reduction was used in the design process [22]. For the grasping force feedback add-on, a list of design requirements was established, considering technical and clinical aspects:

1. The add-on should measure grasping forces between $0N$ and $15N$, which is consistent with reported grasping force levels in laparoscopic procedures involving delicate tissue [23], [24].
2. The add-on should provide effective vibrotactile feedback to the user about the grasping force in three feedback levels (low (no feedback), approaching threshold, exceeding threshold) to ensure easily interpretable feedback [25].
3. The force threshold for feedback should be adjustable for research and development purposes.
4. The add-on should not compromise instrument functionality (i.e. grasping, axial rotation) to prevent loss of surgical performance [26].
5. The add-on should have a diameter equal or less than $50mm$ and a length equal or less than $50mm$ to minimize impact on instrument handling.
6. The weight of the add-on is less than 100 grams to minimize surgeon fatigue [27].

**FIGURE 1.**
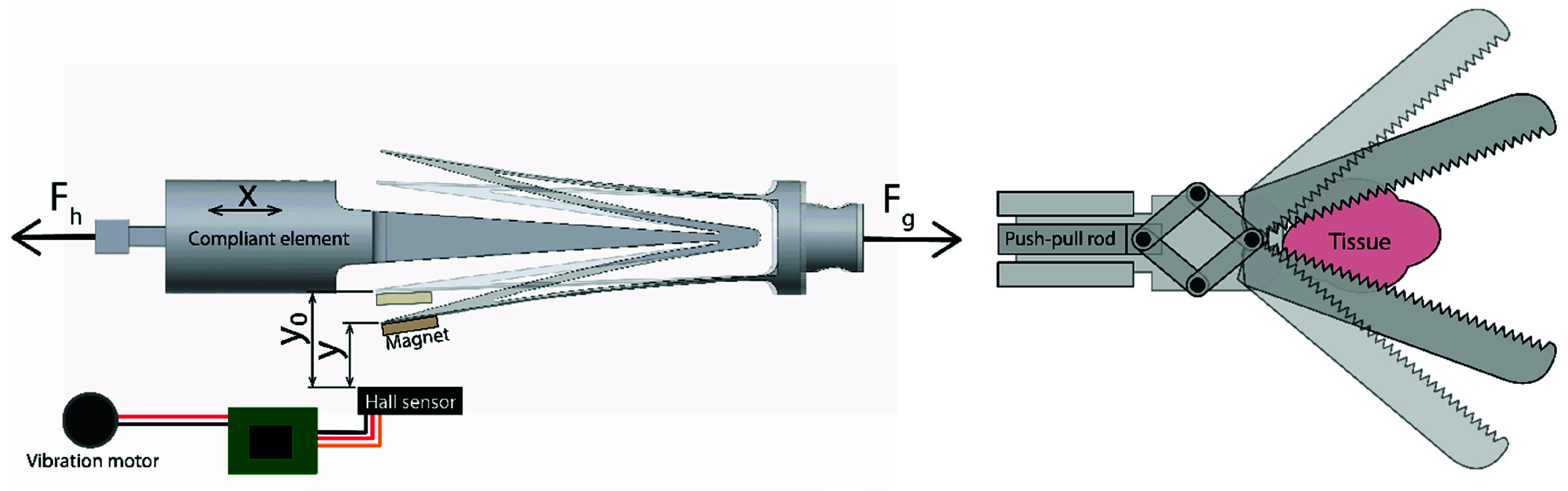
General working principle of the ShaftFlex grasping force compliant measurement system.

To measure grasping forces, the ShaftFlex was developed. The ShaftFlex is a compliant element that deforms when a tensile force is applied to it. It was integrated into a conventional laparoscopic grasper, connecting the instrument handle to the push-pull rod. Fig. 1 shows the ShaftFlex general working principle, consisting of two mounting points on either side and a compliant part in between. The working principle is as follows: a user is exerting force $F_{h}$ on the handle side of the grasper. As a result, a grasping reaction force $F_{g}$ is exerted on the ShaftFlex via the push-pull rod. This causes the compliant part to extend outwards. A magnet is mounted on one of the extending tips, close to a Hall sensor (OH49E, Ouzhou Sensing & Connecting, Nanjing, China). When a grasping force is applied, the magnet moves closer to the Hall sensor, decreasing distance $y$ and increasing the measured magnetic field strength, which in turn increases the sensor’s voltage output.

This voltage is then read by an Arduino-based PCB controller (Arduino, Turin, Italy), which drives a vibration motor (Seeed Studio, Shenzhen, China). The vibration motor in the Shaftflex provides perceivable vibrotactile feedback when a certain force threshold is exceeded. This threshold is adaptable. When the grasper jaws are actuated by a user without grasping any tissue, the ShafFlex functions as a non-deforming part, similarly as the push-pull rod.

The ShaftFlex mounted to the push-pull rod, close to the handle. Moreover, it is a removable add-on. This reduces constraints regarding cleaning, sterilization and sensor size, improving the feasibility and sustainability of the instrument.

### A. Technical Validation

**FIGURE 2.**
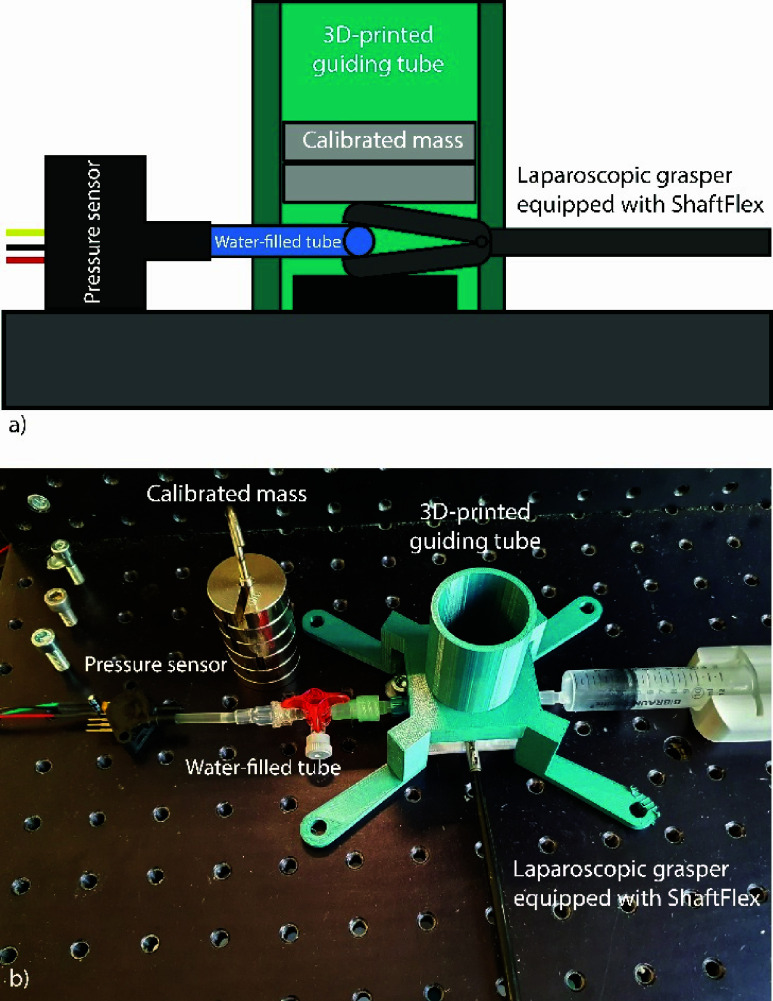
Schematic visualization (a) and photo (b) of the experiment setup for the sensor calibration.

The laparoscopic grasper was calibrated to ensure a correct measurement of the grasping forces. To have a realistic mapping of grasping force on soft tissue to the compliant element, a two-step calibration process was used. First, known masses (in 100 gram increments) were placed on the grasping tip, which compressed a water-filled flexible tube (Fig. 2a and b). The resulting internal pressure was measured using a piezoresistive pressure sensor (MXP5500DP, NXP Semiconductors, Eindhoven, The Netherlands). This allowed us to establish a reference relationship between applied force and internal pressure in the tube. In the second step, the instrument (equipped with the ShaftFlex) grasped the same water-filled tube. During this, the internal pressure and the Hall sensor readings were recorded simultaneously. For both steps, the lowest sensor output was subtracted from all measurements and set to 0.00 V, serving as offset correction. By first establishing the relationship between applied force and internal pressure (first step), and then mapping internal pressure to Hall sensor values (second step), a direct relation between Hall sensor output and grasping force could be derived. Both steps were executed 5 times, and the mean values were used in MATLAB (The MathWorks Inc. Natick, MA) to fit a function mapping between grasping force and Hall sensor output.

### B. Preclinical Validation

**FIGURE 3.**
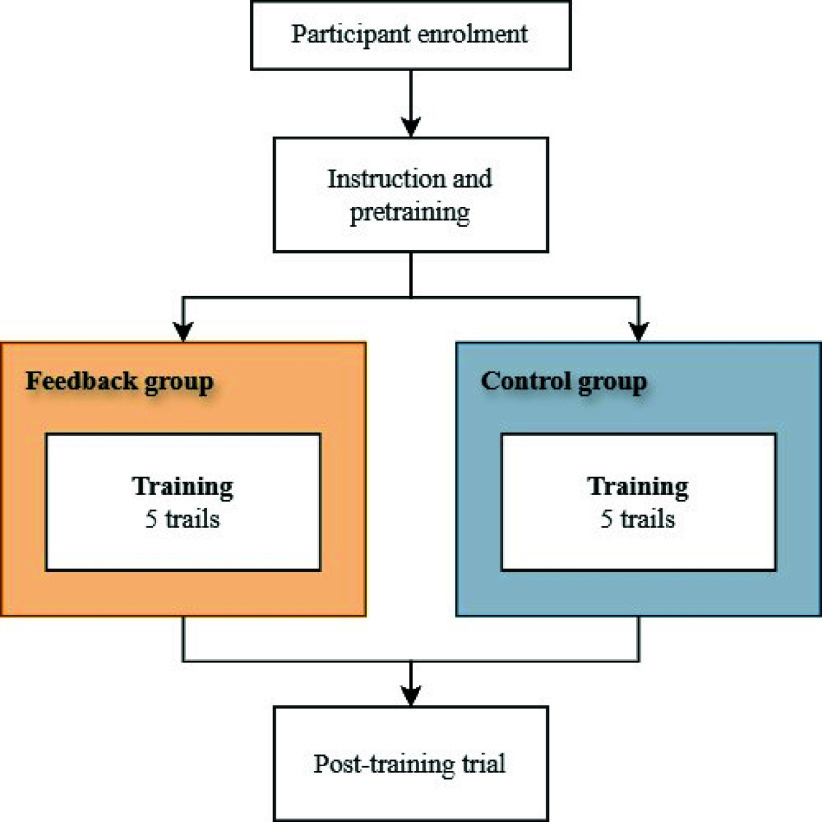
ShaftFlex learning curve study protocol.

Influence on skills acquisition was assessed in a randomized controlled trial. Participants were recruited from the faculty of Mechanical Engineering at the Delft University of Technology. Participants were required to have no prior experience with laparoscopic skills training and surgery. After they had given informed consent, participants were randomly assigned into two groups: a Feedback group and a Control group (Fig. 3). Participants were instructed, after which they completed a pretraining trial to familiarize them with the boxtrainer, the instrument and the task. Thereafter, both groups performed 5 consecutive training trials with the instrument. In the Feedback group, the grasping force feedback was enabled, while in the Control group, the feedback was disabled. For the tasks in the Feedback group, three grasping force feedback levels were applied in the ShaftFlex PCB controller: no feedback when a grasping force of $0N-3N$ was applied, a low intensity vibrotactile feedback for $3N-4N$ and a high intensity vibrotactile feedback for $4N-6N$. Force ranges were divided into three categories (0–3 N, 3–4 N, 4–6 N). These levels were chosen based on the measurement range of the ShaftFlex and the task characteristics, to allow consistent within-study comparisons. After the training trials, both groups performed the same post-training trial without feedback.

**FIGURE 4.**
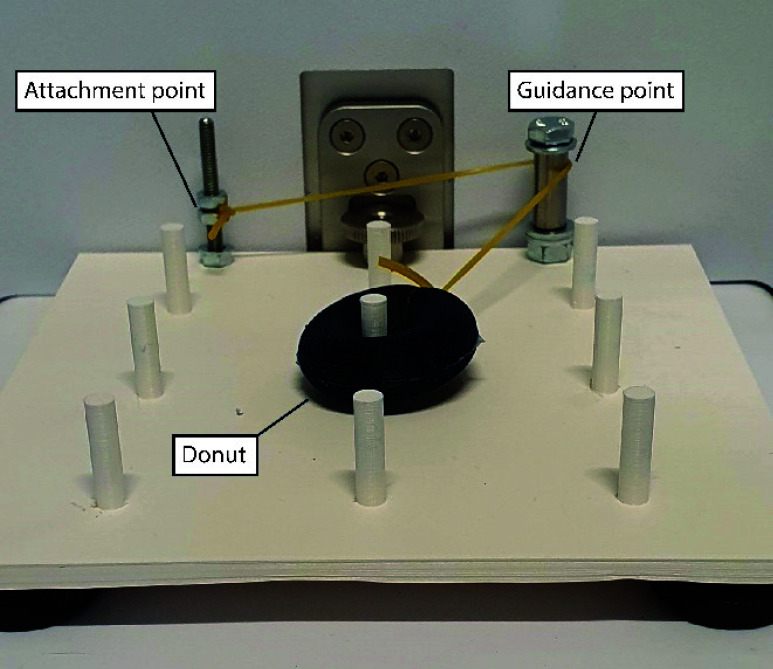
Donut placement training task used in the study. The elastic band is fixed to the task platform at the attachment point and can move freely around the guidance point.

The task was specifically designed to measure the efficacy of the instrument. It consists of a platform with a $3\times 3$ peg grid (Fig. 4). During the task, participants were asked to place a silicone donut with a diameter of 30mm over the pegs. The donut was attached to the task with a short elastic band, pulling on the donut with a force $F_{p}$. This force varied when the donut is moved relative to the attachment point of the elastic band. In this way, the appropriate grasping force, needed to hold the donut when it is moved to a different peg, is constantly changing during task execution. If the grasping force is too low, the donut will slip from the grasper, but if grasping force is too high, the vibrotactile feedback will be activated in the Feedback group. For the study, the Lapron boxtrainer (ForceSense B.V., Delft, The Netherlands) was used, equipped with the ForceSense objective measurement system (ForceSense B.V., Delft, The Netherlands).

### C. Ethical Considerations

The study design was approved under Human Research Ethics Committee (TU Delft HREC) application number 2587. Informed consent was obtained from participants prior to study participation. Data was anonymized to ensure privacy. Participants did not receive any compensation.

### D. Statistics

The data was tested for normal distribution using the Shapiro-Wilk test. Statistical analysis was done using RStudio (Posit Software, Boston, MA). Normal distribution of the differences between the groups could not be assumed, therefore significance of the measured effect within groups was tested with the Wilcoxon signed-rank test, while significance between groups was tested with the Wilcoxon rank-sum test.

### E. Questionnaire

Two expert surgeons ( $> 20$ years of experience) were asked to use the laparoscopic grasper with the ShaftFlex add-on and were interviewed about their experience with the instrument and its potential benefits and challenges of surgery.

## III. Results

### A. Design

**FIGURE 5.**
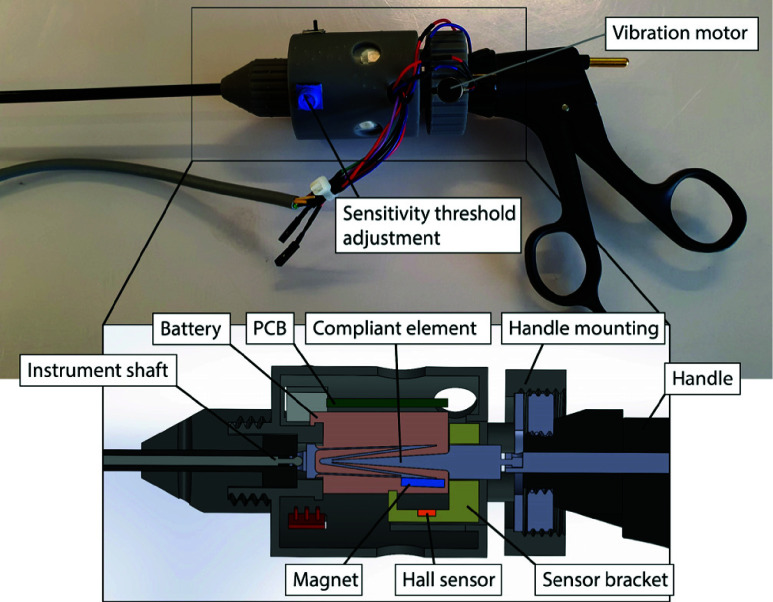
Close-up of the ShaftFlex force measurement unit mounted on the laparoscopic grasper, with a detail cross section of the internal components and mounting to the instrument shaft and handle.

The cylinder-shaped add-on (Fig. 5) fitted on the instrument and did not impede grasping and axial rotation. It has a diameter of 44mm, a length of 50mm and a mass of 98 grams. The housing was 3D-printed from Grey Resin using a Formlabs 3D printer (Formlabs Inc., Somerville, MA, USA).

The electronics of the feedback system involve a Hall sensor, an ATmega328 microcontroller (Microchip Technology Inc., Gresham, OR), a rechargeable 800mAh battery (Cellevia batteries, Shenzhen, China), two vibration motors (Seeed Studio, Shenzhen, China) for the feedback provision and a potentiometer to set the grasping force feedback levels. To ensure sufficient perceptibility of the vibrotactile feedback, the vibration motors used to provide feedback were placed on the proximal side of the add-on (Fig. 5).

### B. Technical Validation

During technical validation, it was observed that the compliant element deformed plastically at a grasping force of 6.4N. Therefore, the ShaftFlex was only validated for grasping forces lower than 6N.

**FIGURE 6.**
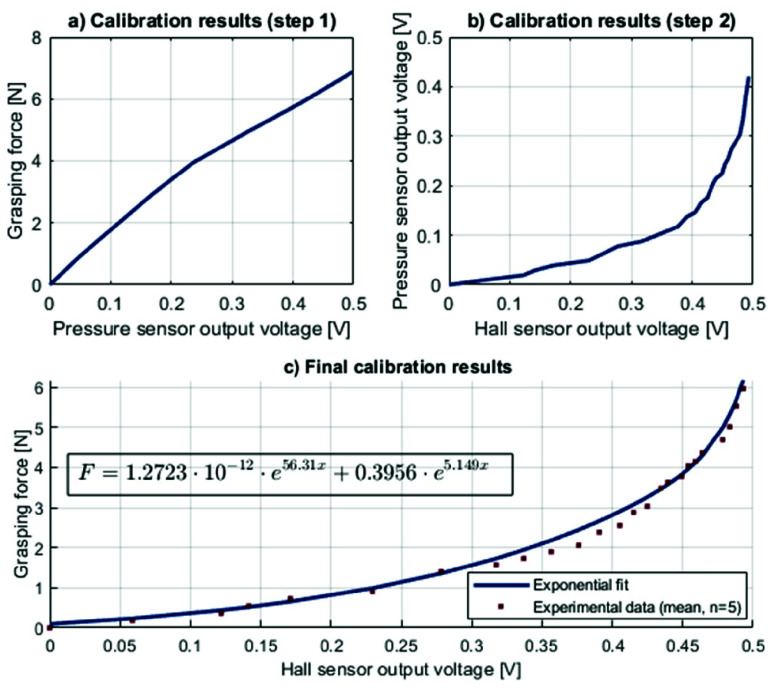
Calibration results.

The results of the two-step calibration process are as follows: the relation between the grasping force-pressure sensor is shown in Fig. 6a. The mean pressure values in the first step increased linearly with applied force, ranging from 0.00 V (offset-corrected, SD 0.014) at 0 N to 0.498 V (SD 0.012) at 6 N, indicating a stable and consistent response. In contrast, the relation between the pressure sensor output and the Hall sensor was non-linear (Fig. 6b). In the second step, the mean Hall values rose from 0.00 V (offset-corrected, SD 0.049) at 0 V to 0.423 V (SD 0.006) at 0.419 V, again showing low variability. Therefore, interpolation of the grasping force and second-order exponential regression of the averaged data yielded a non-linear relation between grasping force and Hall sensor output (Fig. 6c):

\begin{equation*} F_{g}=a\cdot e^{bx}+c\cdot e^{dx}\tag{1} \end{equation*}

With $a=1.2723\cdot {10}^{-12}$, $b=56.31$, $c=0.3956$ and $d=5.149$.

This equation was used in the Arduino script to map Hall sensor input to force levels.

### C. Preclinical Validation

**FIGURE 7.**
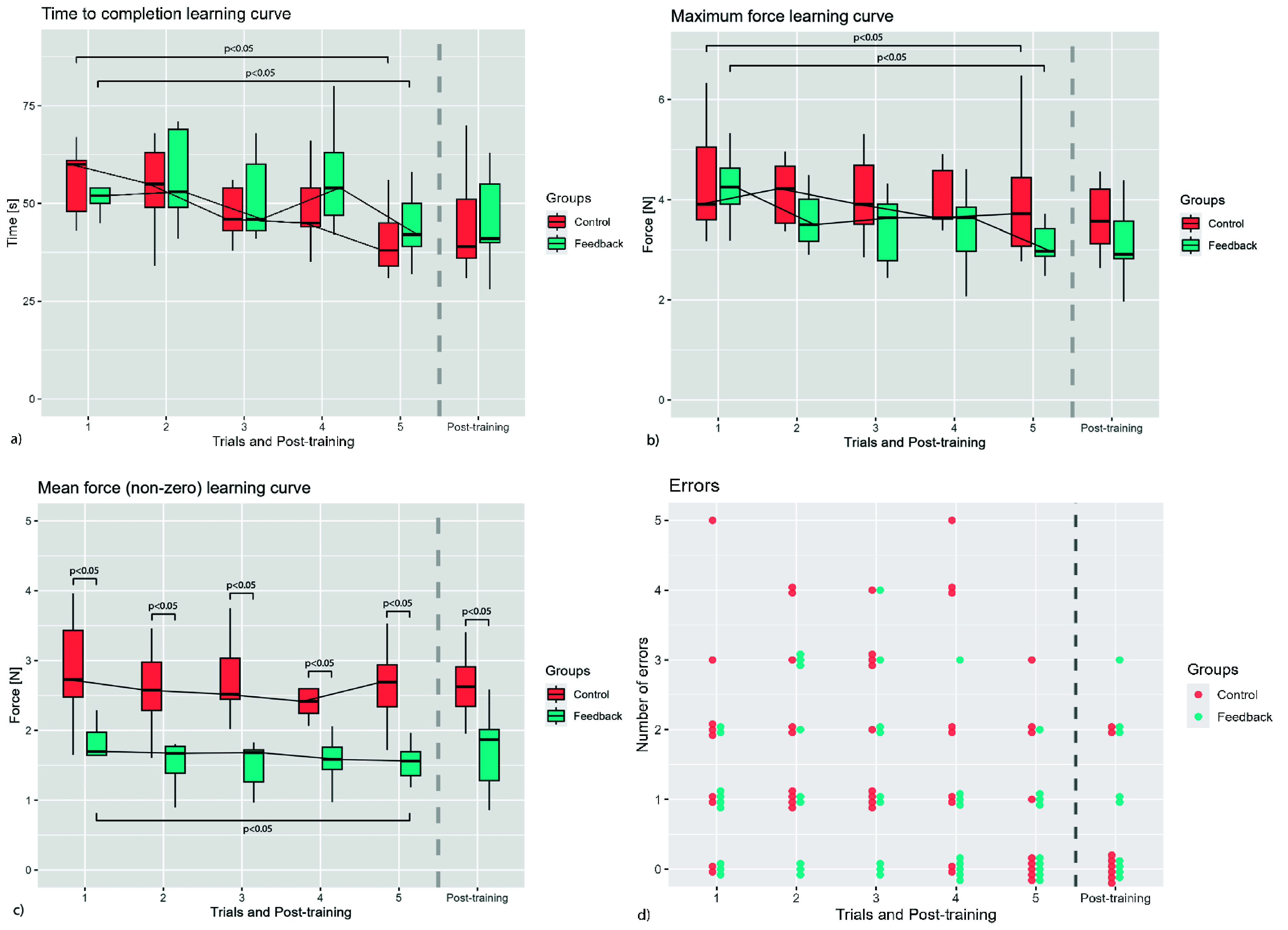
Results of the time to completion (a), maximum grasping force (b), mean force (non-zero) (c) and error measurements (d).

The total number of participants was 18 (7 females and 11 males, mean age 26.6±5.91). All participants had no experience in surgical training. Table 1 shows the descriptive statistics for all trials and the post-training. In Fig. 7a-d, the results of the performance parameters of all five and the post-training trial are shown. For both the Feedback and Control groups, there was a significant difference between the first and last training trial in time to completion (Fig. 7a) as well as in maximum force (Fig. 7b). Regarding non-zero mean grasping force (Fig. 7c), there was a significant difference between the groups for each training trial and the post-training trial. There were no significant differences in slipping errors between the groups or trials (Fig. 7d). The participants were able to apply an appropriate grasping force level, both high enough to prevent slippage and also low enough to prevent tissue damage [5]. Also, they adapted their grasping force to the varying force $F_{p}$ pulling on the donut.

**TABLE 1.** Descriptive Statistics of the Preclinical Validation Results (SD Indicates Standard Deviation).

	Trial 1		Trial 2		Trial 3		Trial 4		Trial 5		Post-training	
	Mean	SD	Mean	SD	Mean	SD	Mean	SD	Mean	SD	Mean	SD
Time (s) Control	55.9	8.6	53.8	11.9	52.4	20.7	48.4	9.5	41.2	9.5	44.7	13.2
Feedback	55.1	13.9	55.9	11.4	51.2	10.4	55.7	12.1	44.1	7.7	48.8	19.0
Errors (n) Control	1.8	1.6	2.1	1.3	2.1	1.2	2.1	1.8	0.9	1.2	1.2	2.3
Feedback	0.8	0.8	1.4	1.3	1.4	1.4	0.7	1.0	0.6	0.7	1.1	1.1
Maximum force (N) Control	4.38	1.05	4.13	0.66	4.26	1.15	4.16	0.97	3.99	1.18	3.79	1.02
Feedback	4.39	0.86	3.56	0.57	3.42	0.72	3.44	0.88	2.99	0.52	3.23	1.03
Mean force (N) Control	2.92	0.75	2.52	0.73	2.69	0.85	2.59	0.75	2.59	0.74	2.54	0.63
Feedback	1.70	0.48	1.55	0.30	1.50	0.32	1.52	0.43	1.55	0.26	1.71	0.56

### D. Questionnaire

Both expert surgeons provided written feedback that an instrument equipped with the ShaftFlex add-on can be a valuable tool in reducing the risk of tissue damage due to excessive grasping force. It was also stated that its benefit might mainly apply to surgery targeting delicate tissue, such as bowel, mesentery, veins and in paediatric surgery. Regarding the vibrotactile feedback characteristics, the surgeons stated that they would prefer a short duration (pulsating) feedback signal over the current continuous feedback. Also, one of the surgeons mentioned that using a potentiometer for adjusting the force level thresholds was not intuitive, because surgeons are unfamiliar with force magnitude estimation. He stated that in a clinical setting, threshold force level adaptation should be limited to one or two settings. All written feedback can be found in supplemental file A.

## IV. Discussion

### A. Design and Technical Validation

The ShaftFlex working principle provided a feasible method to measure grasping forces exerted by a laparoscopic grasper. The compliant element reliably provided vibrotactile feedback for grasping forces up to 6N. Moreover, the add-on enabled grasping force data logging. However, grasping force exceeding 6N resulted in plastic deformation of the compliant element. Therefore, Design requirement 1 was not entirely fulfilled. The compliant element should be redesigned to reduce internal mechanical stress and increase the allowable grasping force. This can be achieved by increasing the flexure thickness or flexure length. As this will alter the relation between $y$-displacement and grasping force, a different magnet and Hall sensor might be needed.

The ShaftFlex was able to provide vibrotactile feedback to the user regarding the applied grasping force in three levels, fulfilling Design requirement 2. An alternative feedback approach could be to provide visual or auditory warning signals. However, previous studies have shown that tactile feedback is more effective than visual or auditory feedback in force perception contexts, as it does not further increase the cognitive load on the visual and auditory channels [28], [29], [30]. Therefore, vibrotactile feedback was chosen as the most promising modality, while acknowledging that future work could still compare these approaches in terms of usability and ergonomic impact. The force threshold for all levels was adjustable (Design requirement 3) using a potentiometer. It should be noted that the force level adjustment was implemented for engineering and validation purposes. However, as pointed out in the expert opinion, surgeons are unfamiliar with adaptation of force feedback thresholds. Therefore, for clinical applications, the appropriate threshold levels should be limited or fixed to prevent unsuitable level selection. Further development steps should involve research and selection of the appropriate threshold level for different surgery types. The instrument’s functions are not impeded by the ShaftFlex, as participants were able to grasp the donut and rotate the shaft, thereby fulfilling Design requirement 4. The ShaftFlex has a diameter of 44mm, a length of 50mm and a mass of 98 grams, fulfilling Design requirement 6 and 7. To strengthen this finding, no comments were given about the instrument size and weight during or after the trials in relation to its functionality.

The compact custom designed PCB provided flexibility for the electronic design. It was programmable using Arduino code via a serial connection, which enabled adaption of the threshold levels and intensity of the vibrotactile feedback. This is beneficial for an iterative design process, allowing changes to the feedback characteristics after the add-on was assembled.

### B. Preclinical Validation

In the comparison of mean (non-zero) force between the Feedback group and the Control group it was found that vibrotactile feedback resulted in significant group differences in all trails $(p< 0.05)$. This is consistent with our hypothesis that during training, grasping with force feedback will reduce the mean grasping force compared to grasping without force feedback.

Secondly, it was hypothesized that when grasping force feedback is removed after training with force feedback, participants will exert higher grasping forces compared to participants that trained without force feedback. Contrary to our expectations, we found that in the post-training group, where feedback was removed, the Feedback group exerted significantly lower mean grasping forces compared to the Control group. Therefore, the second hypothesis is rejected. These results suggest that during training, participants learned to not exceed the grasping force threshold without relying on the feedback signal itself, which is consistent with findings from another study [31]. Grasping forces were suppressed by participants even when the signal was absent. This suggests that vibrotactile feedback might be used to create an improved perceptual framework of safe tissue grasping forces in an intuitive way. For this, it is crucial that training with feedback has a lasting effect. For ShaftFlex to be validated as an effective training tool, it is crucial that the training effect persists in the long term. If the effect does not last, ShaftFlex may still be useful as a clinical device, but its role in training cannot be established. Therefore, further research should specifically investigate the sustainability of grasping force suppression after feedback is removed. Also, future research should explore the ability to discern tissue of different stiffness, as shown in another study [20].

There were no significant differences in slipping errors and time to completion between the Feedback group and the Control group in any trial. This indicates that feedback provided by the ShaftFlex add-on does not increase task difficulty. The donut placement task was specifically designed for this study. Several objective skill parameters (time to completion, mean (non-zero) force and maximum force) showed a significant difference between the first and the last training trial, indicating of a learning curve. However, these learning curves did not plateau. Also, the task can be completed by a novice within a few minutes, showing that the task is a viable tool to assess performance of novices. To prevent slipping, novices had to apply a variable grasping force $F_{g}$, dependent on the elastic force $F_{p}$ pulling on the donut. This suggests that the task might be useful in laparoscopic skills training because often, tissue grasping with laparoscopic instruments involves a combination of pulling and pinching.

### C. Impact

The vibrotactile feedback reduced mean grasping force. This has potential benefits for laparoscopy in general. Also, an important advantage of the ShaftFlex concept is that the sensors are placed in the handle rather than at the grasping jaws. This design minimizes constraints on sensor miniaturization and avoids sterilization challenges at the instrument tip, which are known barriers to clinical translation of cost-effective sensorized instruments. Although the entire instrument must be prepared, cleaned, sterilized in clinical practice, locating the sensing mechanism in the handle reduces exposure of electronic components to patient-contacting regions and eliminates the need for miniaturization of the sensing element within the jaws. It further removes the necessity of integrating additional wiring for communication between the sensor and the handle. This avoids small cavities that are difficult to clean and sterilize, simplifying the overall design and improving maintainability compared to tip-integrated systems. This strengthens the clinical relevance of the design and distinguishes ShaftFlex from instruments that require sensor integration at the tip. Nonetheless, the add-on does increase instrument cost. Therefore, the add-on should only be applied when there is a significant patient outcome benefit, for example when the surgeon targeting delicate tissue, increasing the risk of tissue perforation. For this reason, cholecystectomy, bowel surgery or paediatric surgery might benefit the most from added grasping feedback, according to the expert opinion obtained in the questionnaire. Also, the ShaftFlex concept might be beneficial in robotic surgery, where force feedback is mostly lacking. As the ShaftFlex provides a technically feasible solution for grasping force measurements, it is especially interesting to integrate with cost effective, sustainability-oriented robot-assisted surgery systems such as the AdLap RS [32]. The force ranges used in this study served as a pragmatic categorization and did not affect the primary finding that vibrotactile feedback reduces mean grasping force. For clinical application, these thresholds would need to be re-evaluated and validated against procedure-specific safety criteria.

In addition to its clinical implications, ShaftFlex also provides continuous grasping force data that may enhance laparoscopic training. The ShaftFlex provided continuous grasping force data. In an earlier study, it has been proven that parameters such as time to completion and forces exerted on a laparoscopic task can be used to accurately identify participant skill level [7], [33]. As applying excessive grasping force is a potential indicator for low skill level as well [34] and a source of complications during surgery [2], grasping force measurements can be used additionally to the aforementioned objective skill parameters to provide feedback and to compare training performance between training trials and trainees. Therefore, laparoscopic skills training can be enhanced by using the grasping force data provided by the ShaftFlex add-on.

### D. Limitations

It has been shown that laparoscopic instrument grasping forces measured in the instrument shaft are dependent on the opening angle of the grasper jaws, linkage geometry [35] and the distance from the jaw pivot point to the point in the grasper at which the tissue is grasped. The calibration did take the linkage geometry into account, and in the donut task used in this study, tissue was grasped with a relatively large portion of the jaw surface, which likely reduced the effect of variation in grasping location. However, in clinical practice, where contact areas may be smaller, this variation could have a greater impact. Jaw opening angle was not included in the calibration. Accurate estimation of absolute grasping forces would require incorporation of this parameter. This study holds value to assess relative force differences between trials and participants due to the nature of the task, where the jaw opening angle is mostly dictated by the shape of the donut. However, for accurate grasping force measurement, jaw opening angle should be integrated into the calibration. For future applications, integration of a jaw opening angle sensor (e.g., in the handle, which directly translates to jaw aperture) would be needed while maintaining the design principle of avoiding sensors in the tip.

A limitation of the ShaftFlex is that force measurements at the shaft include the effects of internal friction and backlash, thereby reducing the fidelity of the transmitted haptic information. As a result, not all grasping force or tissue stiffness information is preserved. Nevertheless, as the results show, the system still provides meaningful feedback that can help suppress excessive grasping forces. Also, by placing the sensors outside the tip, the design avoids the aforementioned miniaturization and sterilization challenges.

Although the number of trials was limited to five, previous work has shown that a small number of repetitions can already provide reliable information on objective performance parameters [7], [36]. Nevertheless, future studies with extended training sessions and larger datasets are warranted to assess long-term learning effects and performance stability in more detail.

### E. Further Research

In this study, it has been shown that immediate force level feedback reduces applied grasping forces during training, even when feedback is removed. However, it is not known if this effect remains after multiple trials without feedback. If this is the case, the ShaftFlex can be used in laparoscopic skills training to quickly educate trainees on the appropriate amount of grasping force. To investigate long-term effects of grasping force level feedback after feedback is removed, the study should be replicated with longer training series and delayed retention tests to determine whether the observed reduction in grasping force persists over time, thereby confirming the long-term effectiveness of ShaftFlex as a training tool.

## V. Conclusion

The ShaftFlex, an add-on for laparoscopic graspers, was developed to provide instantaneous vibrotactile grasping force feedback, with the primary effect of reducing excessive grasping forces in minimally invasive surgery. As a secondary conclusion, it may also offer a new method to enhance safe tissue grasping during basic laparoscopic skills training. The ShaftFlex reliably provided feedback for grasping forces up to 6N. A redesign of the compliant element is needed to increase the maximum allowable grasping forces. In the participant study, it was shown that laparoscopic skills training with grasping force feedback reduces the grasping force compared to training without grasping force feedback. Moreover, when feedback was removed in the Feedback group, mean (non-zero) force was still lower compared to the Control group, suggesting that participants learned to not exceed the grasping force threshold without relying on the feedback signal itself. More research is needed to investigate the long-term effects of training with vibrotactile grasping force feedback.

## VI. Conflicts of Interest

The authors have no conflicts of interest to declare.

## VII. Data Availability

The dataset from this study is available at 4TU Research Data Repository (DOI: 10.4121/8b5eadbd-bb15-43c2-bdf7-58208c314049)

## VIII. Author Contributions

Jan-Willem Klok worked on the ShafFlex design, provided the technical and pre-clinical validation study design, the results interpretation, the discussion with its clinical impact, provided the manuscript draft and coordinated the coauthor review and submission process. Yannick Smits provided the detailed design of the ShaftFlex, executed the technical and pre-clinical validation and contributed to the manuscript draft. Roelf Postema provided clinical input during the validation phase and reviewed the manuscript draft. Asþor T. Steinþorsson contributed to the initial design and reviewed the manuscript draft. Jenny Dankelman reviewed the manuscript draft. Tim Horeman provided technical input during the course of the project, contributed to results interpretation and reviewed the manuscript draft.

## Supplementary Materials

Supplementary Materials
