# Supplementary material for: Design and Validation of a Grasping Force Measuring Vibrotactile Feedback Add-On for Laparoscopic Instruments
Source: IEEE J Transl Eng Health Med. 2025 Nov 28;14:1–10. doi: 10.1109/JTEHM.2025.3638856 (PMC12772989; doi:10.1109/JTEHM.2025.3638856)
Supplement: Supplementary Materials [file supp1-3638856.docx]

Expert opinion 1: The concept of having a feedback system in an add-on form factor he was very enthusiastic about. He believes that it is better to have modular technologies instead of replacing functioning instruments. Expert 1 liked the vibrotactile feedback, stating that it can help to prevent high grasping forces. He suggested a Bluetooth connected LED module that you can place near the screen of the trainer so that it is in your field of view. About the vibrotactile feedback, he stressed that the signal should be short. While the feedback signal is being given, the sensors in the hand are occupied by that signal and control of the instrument can be impaired. Therefore, use pulses and keep the feedback duration short.

Expert opinion 2: This expert mentioned Bluetooth or other wireless connection to get rid of the cables attached to the add-on. Furthermore, he liked the vibrotactile feedback on the device. He suggested making the vibration signal more phased. He finds the current feedback signal not suitable for use in surgery, however, for the training tasks performed in this study, his opinion is that it works well. The grasping force feedback level adjustment may not be very suitable for use in surgery, because surgeons are not familiar with this kind of adjustment. He suggested having one or two preset feedback level settings.
